# The Role of Oxidative Inactivation of Phosphatase PTEN and TCPTP in Fatty Liver Disease

**DOI:** 10.3390/antiox12010120

**Published:** 2023-01-03

**Authors:** Thang Nguyen Huu, Jiyoung Park, Ying Zhang, Hien Duong Thanh, Iha Park, Jin Myung Choi, Hyun Joong Yoon, Sang Chul Park, Hyun Ae Woo, Seung-Rock Lee

**Affiliations:** 1 Department of Biochemistry, Department of Biomedical Sciences, Research Center for Aging and Geriatrics, Research Institute of Medical Sciences, Chonnam National University Medical School, Gwangju 61469, Republic of Korea; 2 BioMedical Sciences Graduate Program (BMSGP), Chonnam National University Medical School, Hwasun 58 128, Republic of Korea; 3 College of Pharmacy, Graduate School of Pharmaceutical Sciences, Ewha Womans University, Seoul 120-750, Republic of Korea; 4 Department of Cell Biology, School of Medicine, Jiangsu University, Zhenjiang 212013, China; 5 Department of Anatomy, Chonnam National University Medical School, Gwangju 61469, Republic of Korea; 6 The Future Life and Society Research Center, Advanced Institute of Aging Science, Chonnam National University, Gwangju 61469, Republic of Korea

**Keywords:** ALD, NAFLD, PTEN, TCPTP, ROS, redox regulation

## Abstract

Alcoholic liver disease (ALD) and nonalcoholic fatty liver disease (NAFLD) are becoming increasingly prevalent worldwide. Despite the different etiologies, their spectra and histological feature are similar, from simple steatosis to more advanced stages such as steatohepatitis, fibrosis, cirrhosis, and hepatocellular carcinoma. Studies including peroxiredoxin knockout models revealed that oxidative stress is crucial in these diseases, which present as consequences of redox imbalance. Protein tyrosine phosphatases (PTPs) are a superfamily of enzymes that are major targets of reactive oxygen species (ROS) because of an oxidation-susceptible nucleophilic cysteine in their active site. Herein, we review the oxidative inactivation of two tumor suppressor PTPs, phosphatase and tensin homolog deleted on chromosome 10 (PTEN) and T-cell protein tyrosine phosphatase (TCPTP), and their contribution to the pathogenicity of ALD and NAFLD, respectively. This review might provide a better understanding of the pathogenic mechanisms of these diseases and help develop new therapeutic strategies to treat fatty liver disease.

## 1. Introduction

The liver is the second-largest organ in the body and has functions in metabolism, excretion, and immunology [1]. Fatty liver disease is defined by excessive fat accumulation that exceeds 5% of the liver weight and is becoming increasingly common worldwide [2]. Alcoholic liver disease (ALD) and nonalcoholic fatty liver disease (NAFLD) are two subgroups of fatty liver disease [3]. Heavy alcohol consumption is the only cause of ALD, whereas a high-fat diet (HFD), obesity, and insulin resistance (IR) are major factors that contribute to NAFLD. Fatty liver disease starts from a simple steatosis, steatohepatitis, fibrosis, or cirrhosis and can develop into hepatocellular carcinoma (HCC) depending on the disease progression [4]. Some patients with steatohepatitis can develop HCC without cirrhosis [5]. Two early stages of fatty liver disease are reversible, whereas advanced stages are irreversible and display a poor prognosis.

Protein tyrosine phosphatases (PTPs) are a superfamily of protein phosphatases that play a key role in controlling signaling transductions [6]. In contrast to protein tyrosine kinases, PTPs catalyze the removal of phosphate groups from phosphorylated tyrosine residues [6]. PTPs are divided into two classes: the classical tyrosine-specific phosphatases and the dual-specificity phosphatases [6,7]. Among dual-specificity phosphatases, the tumor suppressor, phosphatase and tensin homolog deleted on chromosome 10 (PTEN), in addition to the main function of lipid phosphatase of phosphatidylinositol (3,4,5)-trisphosphate (PIP3), can dephosphorylate tyrosine phosphate and other substrates such as threonine phosphate and serine phosphate [8,9]. Hepatocyte-specific PTEN deficiency promotes the phenotypes of non-alcoholic steatohepatitis (NASH) and HCC, suggesting the important role of PTEN in lipogenesis, glucose metabolism, and tumorigenesis [10]. The tyrosine-specific PTP class consists of receptor-like and non-transmembrane proteins [6]. T-cell protein tyrosine phosphatase (TCPTP), also known as protein tyrosine phosphatase nonreceptor type 2 (PTPN2), is a well-known non-transmembrane PTP that is cloned from a peripheral human T cell cDNA library [11]. Homozygous TCPTP deficiency has been shown to regulate hepatic gluconeogenesis and insulin signaling [12]. Other studies have indicated that TCPTP also functions as a tumor suppressor [13,14].

Accumulating evidence on ALD and NAFLD pathology reveals that oxidative stress is one of the key factors in disease onset and progression [15,16,17]. Reactive oxygen species (ROS) are over-produced in various ways, including mitochondrial dysfunction, endoplasmic reticulum (ER) stress, and nicotinamide adenine dinucleotide phosphate (NADPH) oxidase (NOX) activity, resulting in an imbalance in redox homeostasis [18]. However, the molecular targets of ROS and underlying mechanisms have not been completely understood. PTPs are important targets of intracellular oxidants such as H_2_O_2_ [19]. All PTPs contain a conserved cysteine residue in the signature catalytic site motif ([I/V] HCXXGXXR [S/T]—X is any amino acid residue) that is essential for catalytic activity at low pKa [6,20,21]. Low pKa promotes the nucleophilic function of the cysteine residue but also renders it highly susceptible to specific oxidation by various ROS, which subsequently prohibits the nucleophilic catalytic function and inhibits the PTP activity. According to the type of oxidation, the nucleophilic cysteine at the active site of PTPs can be oxidized to sulfenic acid (-SOH) or, to a greater extent, to sulfinic (-SO_2_H) or sulfonic (-SO_3_H) acid [6]. Various antioxidant systems can reduce sulfenic acid, whereas the higher levels of oxidation are irreversible and decrease protein levels [6]. Studies have shown that both PTEN and TCPTP are oxidized by ROS and that their oxidative inactivation facilitates fatty liver disease progression. Herein, the pathogenic mechanisms of oxidative inactivation of PTEN and TCPTP in ALD and NAFLD, respectively, are reviewed.

## 2. Oxidative Stress and Peroxiredoxins in ALD and NAFLD

### 2.1. ROS Sources

ROS are produced under physiological conditions and serve as signaling molecules that participate in cellular pathways, such as cell survival, proliferation, and differentiation [22,23]. ROS are chemically classified into free radicals and nonradicals. Free radicals are superoxide anion radicals (O_2_^•−^) and hydroxyl radicals (HO^•^), whereas the most well-known nonradical is H_2_O_2_ [16,23]. However, increased ROS production under pathological conditions facilitates an imbalance in redox homeostasis, triggering cellular damage and oxidative stress via enzyme inactivation, protein denaturation, DNA damage, and lipid peroxidation [18,23]. ROS also has catastrophic effects in both ALD and NAFLD [24,25,26].

ROS generators comprise different organelles, including the endoplasmic reticulum (ER), mitochondria, and peroxisomes; oxidative enzymes, including NOX, xanthine oxidase, aldehyde oxidase, and cyclo-oxygenase; cytochrome P450 2E1 (CYP2E1); and environmental factors, including smoke, UV light, radiation, and certain drugs, such as antibiotics (nitrofurantoin) and methotrexate [16,27]. Cellular defense systems, such as catalase, glutathione peroxidase (Gpx), peroxiredoxin (Prx), thioredoxin (Trx), glutaredoxin (Grx), glutathione (GSH), and superoxide dismutase, safeguard cells and maintain redox homeostasis [27,28].

### 2.2. Oxidative Stress in ALD

Oxidative stress is one of the key drivers of the progression of ALD. ROS are excessively produced upon alcohol consumption, leading to the onset and progression of ALD. Research on the metabolism of ethanol in the liver is very critical for understanding the generation of ROS. The gastrointestinal tract is responsible for the absorption of ethanol, which is primarily metabolized in the liver; however, 3–5% is secreted through other means, such as breath, urine, and sweat [29,30]. In the liver, ethanol metabolism is mediated by three main oxidative metabolic pathways: the alcohol dehydrogenase (ADH) system in the cytoplasm, the microsomal ethanol oxidation system (MEOS), and the catalase system [31]. These systems are driven by ADH and nonspecific enzymes, such as CYP2E1 and catalase, which oxidize ethanol into aldehyde [32,33]. Since aldehydes are highly toxic and carcinogenic, they are rapidly oxidized in the mitochondria by aldehyde dehydrogenase 2 (ALDH2) into acetate. The tricarboxylic acid cycle converts acetate into carbon dioxide and water [32]. Under normal conditions, most of the ethanol consumed is metabolized by ADH in the cytoplasm, and a small amount is metabolized by catalase; however, MEOS activity increases when ethanol concentration is increased due to chronic consumption [34]. In addition to oxidative metabolic pathways, less than 0.1% of the ethanol goes through nonoxidative metabolism, generating phosphatidylethanol and fatty-acid ethyl esters. These ethanol metabolites can be used as biomarkers for the diagnosis of liver diseases [35].

ROS are overproduced upon ethanol consumption via the MEOS system and mitochondrial dysfunction. It has been reported that the activation of MEOS increases during chronic alcohol consumption due to the increased stabilization of CYP2E1 [36], which plays an important role in alcohol oxidation and is a member of the cytochrome P450 family. Ethanol is oxidized by CYP2E1, is catalyzed into aldehyde, and is accompanied by the conversion of NADPH and O_2_ to NADP^+^ and H_2_O, which generates ROS. ROS are excessively produced and cause cellular damage during oxidization of ethanol by CYP2E1 [17]. Additionally, alcohol consumption leads to mitochondrial dysfunction via changes in structure and function, which is associated with ROS generation and enlargement of the hepatic mitochondria. The mitochondrial ETC mediates ROS overproduction upon alcohol consumption. NADH is excessively generated, leading to alterations in the NADH/NAD^+^ ratio, which subsequently decreases the ETC and facilitates the formation of O_2_^•−^ and HO^•^ [37]. Mitochondrial-dysfunction-mediated ROS overproduction results in mitochondrial DNA damage, formation of lipid peroxidation products, such as malondialdehyde and 4-hydroxynonenal (4-HNE), and ROS-induced apoptosis [37].

### 2.3. Oxidative Stress in NAFLD

NAFLD pathogenesis is characterized by two hypotheses: the “two-hit” hypothesis and the “multiple-hit” hypothesis [15,38,39]. The “two-hit” hypothesis states that the first hit causes steatosis to arise due to increases in the expression of lipogenic genes and decreases in free fatty acid (FFA) deterioration, which results from IR, a sedentary lifestyle, and poor nutritional habits. The second hit causes the promotion of oxidative stress due to ROS overproduction, leading to various insults, such as inflammation and apoptosis, which are typical for NASH and fibrosis [15,38]. However, the “multiple-hit” involves multiple factors that coincide, contributing to NAFLD [39]. In addition, NASH is not necessarily a consequence of hepatic steatosis but can be the initiation of liver injury [40]. FFA overload from sources in an HFD, non-esterified fatty acids, and de novo lipogenesis is the most direct cause for the onset of NAFLD-NASH. IR promotes the generation of adipokines and inflammatory cytokines in adipocytes [41]. Concurrently, the lipotoxicity caused by lipid accumulation promotes oxidative stress and affects the physiological functions of the mitochondria and ER [42]. Cumulatively, oxidative stress is considered the most important element in the initiation and progression of NAFLD and NASH [43].

Disequilibrium in lipid metabolism induces oxidative stress via the overproduction of ROS, which affects ROS generators, including the mitochondria, ER, and NOX. In NAFLD, mitochondria have been well-studied for their role in lipid metabolism and are one of the most important sources of ROS. In addition, the participation of the ER and NOX has been described, but the degree of involvement remains unclear. In hepatic cells, the mitochondria adapt to the increase in FFA intake using fatty acid oxidation (FAO), electron transport chain (ETC) activity, and oxidative phosphorylation efficiency [44]. Most studies show preserved or increased FAO via the upregulation of β-oxidation in the early stages of NAFLD [45,46,47]. FAO is a compensatory response to the increase in FFA intake. However, studies have shown the downregulation of ETC activity in NASH [48,49]. The asymmetry in the activities of mitochondrial FAO and ETC induces enhanced electron donors in the ETC and simultaneously boosts electron escape from this pathway, increasing ROS generation [50].

### 2.4. The Crucial Role of ROS and Peroxiredoxins in Pathogenesis of ALD and NAFLD

The antioxidant enzymes, Prxs, are thiol-dependent peroxidases that function as intracellular antioxidants to reduce H_2_O_2_, alkyl hydroperoxides, and peroxynitrite into water and alcohol, thus maintaining cellular redox homeostasis [51,52,53,54]. Upon their initial discovery in yeast in the mid-1990s, Prxs were underrated compared with other defensive enzymes, such as catalase and Gpx; however, later evidence revealed the major role Prxs play in scavenging cellular peroxides [54,55]. Prxs contain a conserved peroxidatic cysteine (CP) in the NH2 terminus that is susceptible to oxidation by H_2_O_2_ and confers a defensive function against toxic peroxides. Most Prxs have an additional resolving cysteine (CR) in the COOH terminus; Prxs are divided into three classes based on this cysteine: the 2-Cys, atypical 2-Cys, and 1-Cys subfamilies [56]. In addition, six isoforms are found among mammalian Prxs, named Prxs I to VI. Prxs I–IV are 2-Cys Prxs, whereas Prxs V and VI are atypical 2-Cys and 1-Cys Prxs, respectively [56]. Prxs I and II localize to the cytosol and nucleus. Prx III is directed toward the mitochondria, since it contains a mitochondrial targeting signal (MTS) at the N-terminal region. Prx IV is the only Prx that localizes to the ER and extracellular space. Similar to Prx III, Prx V is found in the mitochondria and contains an MTS; however, it also localizes to peroxisomes and the cytosol. Lastly, Prx VI is primarily present in the cytosol [56]. Accumulating evidence shows that mammalian Prxs I–IV tend to scavenge H_2_O_2_, whereas Prxs V and VI reduce alkyl hydroperoxides and peroxynitrite. Prxs have been shown to regulate the ROS-scavenging functions and redox regulation of PTEN [57].

Research on Prx knockout has emphasized the importance of oxidative stress in ALD and NAFLD, with mitochondrial ROS being a significant contributor. Pyrazole is known as a substrate of CYP2E1 and is used to study CYP2E1-induced oxidative stress [58]. A previous study showed that pyrazole exposure induced mitochondrial ROS accumulation because of increased CYP2E1 in mitochondria. Interestingly, an increase in Prx III hyperoxidation (sulfinic Prx III) was observed in vitro, and Prx III knockout mice showed increases in lipid peroxidation, liver injury, and apoptosis [59]. This finding suggested the importance of Prx III in the protection of oxidative-stress-induced liver injury. In our recent study, Prx III knockout mice had exacerbated development of alcoholic steatosis, which was induced by ethanol consumption [60]. Prx III deficiency increased lipid accumulation, levels of serum aspartate transaminase, alanine aminotransferase, and liver triglyceride; however, lipid peroxidation was comparable to that of the control group. Notably, Prx III knockout-induced hepatic steatosis was mediated by PTEN oxidation, which resulted in Akt hyperactivation and triggered lipogenesis and adipogenesis [60]. Hence, it was thought that Prx III acted as an ROS scavenger and protected against ethanol-administration-induced PTEN oxidative inactivation.

The role of Prx V in regulating adipogenesis and NAFLD has been widely reported [61,62]. Prx V and ROS were demonstrated to play a vital role in adipogenesis, as indicated by their upregulation in cells that were treated with insulin. In addition, Prx V overexpression inhibited adipogenesis by decreasing adipogenic protein levels, including those of peroxisome-proliferator-activated receptor gamma (PPARγ), CCAAT-enhancer-binding protein alpha (CEBPA), adipocyte protein 2 (aP2), and glucose transporter type 4 (GLUT4) [61]. Prx-V-deficient mice fed an HFD exhibited ROS generation and obesity via increased body weights and fat pads compared with WT mice. Prx V deletion also remarkably increased the expression of adipogenic genes in white adipose tissue [61]. These findings suggest that Prx V is important in regulating adipogenesis-mediated obesity induced by an HFD. Furthermore, a study concluded that Prx V plays an important role in obesity-induced NAFLD [62]. Treatment of the FFA-induced, ROS-mediated, hepatic steatosis increased Prx V expression. Prx V overexpression enhanced the phosphorylation of AMP-activated protein kinase (AMPK) and acetyl-CoA carboxylase, which suppressed the expression of lipogenic genes, such as those encoding sterol regulatory element-binding protein 1 (SREBP1) and fatty acid synthase (FAS). Prx V knockdown showed the opposite effect and resulted in the accumulation of lipid droplets. Prx V knockout mice also displayed HFD-associated hepatic steatosis with increased lipogenesis proteins [62]. These results support the protective effects of Prx V against mitochondrial ROS overproduction in HFD-induced NAFLD.

## 3. Oxidative Inactivation of PTPs in Fatty Liver Disease

### 3.1. PTEN and ALD

#### 3.1.1. PTEN and Its Redox Regulation

PTEN is a well-known tumor suppressor that is frequently deleted in various cancers [63,64]. This 403-amino acid protein contains multiple domains, including an N-terminal phosphatidylinositol 4,5-bisphosphate (PIP2)-binding domain, a phosphatase domain, a C2 domain, a carboxy-terminal tail domain, and a PDZ-binding motif, which helps it act as a protein-lipid phosphatase, dual-specific protein phosphatase, or scaffold protein [65]. PTEN is mainly considered a lipid phosphatase that targets PIP3, a component of the lipid membrane. PTEN dephosphorylates PIP3 into PIP2, suppressing the PI3K/Akt pathway, a significant pathway for cell growth, proliferation, and differentiation [66,67]. PI3K/Akt is also involved in regulating lipid metabolism via SREBP1c [68]. PTEN acts as a dual-specificity protein phosphatase that targets phosphorylated tyrosine, serine, and threonine residues [69]. As a protein phosphatase, PTEN can suppress tumors by dephosphorylating residues on itself or other substrates, such as focal adhesion kinase 1 (FAK1), cAMP-responsive element-binding protein 1 (CREB1), proto-oncogene tyrosine-protein kinase Src (c-Src), and insulin receptor substrate-1 (IRS-1) [70,71,72,73]. In addition to functioning in a PI3K-dependent manner, PTEN exerts its role in tumor suppression by acting as a scaffold protein in the nucleus and cytoplasm, independent of PI3K and the PI3K/Akt axis [69].

**Figure 1:**
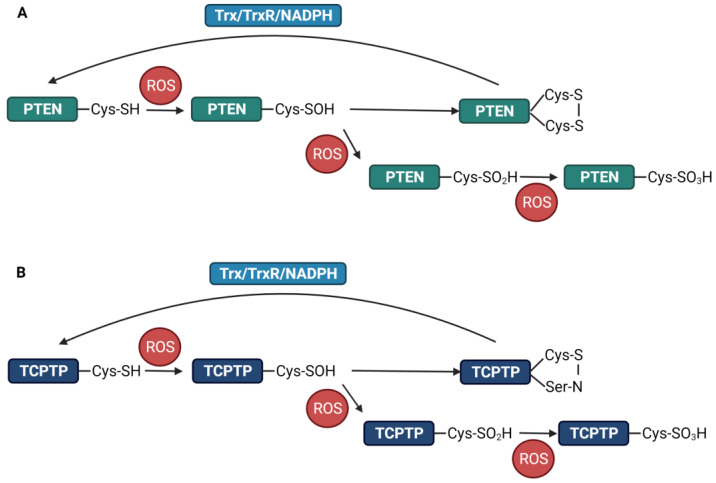
Redox regulation of PTEN and TCPTP by ROS. (**A**). PTEN is reversibly oxidized by ROS and forms a disulfide bond between two cysteine residues. The Trx/TrxR/NADPH system is responsible for the reduction of oxidized PTEN. PTEN may undergo hyperoxidation upon exposure to high levels of ROS. (**B**). TCPTP oxidation into a reversible -SOH is thought to produce a sulfenamide with an adjacent nitrogen atom. The Trx system also plays a key role in reducing oxidized TCPTP. An amount of TCPTP undergoes hyperoxidation into irreversible -SO_2_H and -SO_3_H.

The catalytic domain of PTEN contains cysteine residues at positions 71, 83, 105, 124, and 136 [74]. Among these, Cys^124^ is most susceptible to oxidation by intracellular oxidants. Unlike TCPTP, when ROS inactivate PTEN at Cys^124^, a disulfide bond is formed with the neighboring cysteine, preventing irreversible inactivation by other oxidized species [74]. Studies have shown that this disulfide bond is formed between Cys^124^ and Cys^71^ residues (Figure 1A) [60,74,75]. Following the reversible oxidation of PTEN by H_2_O_2_, oxidized PTEN is reverted to the reduced form by intracellular antioxidant systems, in which the Trx system is thought to play the main role. The disulfide bond is reduced by intracellular antioxidants, such as Trx and GSH, reverting PTEN to its active form [76,77,78]. The reduction of oxidized PTEN by Trx is more effective than that by Grx or GSH in vitro. Trx interaction with PTEN has been demonstrated using co-immunoprecipitation, which supports the Trx reductase activity [74]. PTEN oxidation was determined after a five-minute exposure to H_2_O_2_ and lasted for up to 60 min of incubation. Furthermore, the accumulation of the homodimers of Trx inactive forms had a tendency similar to that of H_2_O_2_-induced PTEN oxidation [75], suggesting that Trx physiologically functions as a monomer in a reduced form and is vulnerably oxidized to form dimers or oligomers [79,80]. Trx dimerization and oligomerization also occurred upon exposure to organic hydroperoxides [76,77], resulting in the irreversible oxidation of PTEN. Overall, the redox regulation of PTEN is tightly associated with the Trx system.

#### 3.1.2. Oxidative Inactivation of PTEN in ALD

PTEN expression in NAFLD and ALD has been investigated via numerous studies. PTEN levels were decreased in rat livers with HFD-induced hepatic steatosis and human HepG2 cells treated with unsaturated fatty acids [81]. This decrease was mediated by mTOR/NF-κB-induced microRNA-21 (miR-21) upregulation, which induced PTEN degradation via miR-21 binding [82]. In addition, PTEN expression declined in 43 out of the 105 HCC tissue samples, indicating that it was associated with tumor progression and decreased overall survival [83]. A recent study has shown that PTEN expression was downregulated in 50.3% of HCC tissues [84]. PTEN knockout was used to investigate its role in NAFLD and HCC. A previous study revealed that 40-week-old hepatic PTEN knockout mice displayed the morphology of NASH, which was mediated by increased PPARγ, SREBP1c, and β-oxidation levels, during histological examinations [10]. Furthermore, 66% of 74- to 78-week-old PTEN knockout mice showed hepatic tumors, which were confirmed to be HCC [10]. This finding suggests a significant role of PTEN in lipogenesis and tumorigenesis. Another study used four-month-old mice with a hepatic PTEN deficiency and showed hepatic steatosis, increased insulin sensitivity, and glucose uptake with an increased de novo lipogenesis, glycolysis, and glucose infusion rate [85]. Interestingly, there was a decline in white adipose tissue depots, which was confirmed by the downregulation of FAS and esterification. These data revealed the crosstalk between the liver, muscles, and white adipose tissue in PTEN-deficiency-mediated hepatic steatosis with elevated insulin sensitivity, increased glucose tolerance, and diminished adiposity [85]. PTEN knockout by CRISPR/Cas9 system showed an elevation in Akt phosphorylation and lipid accumulation in the liver [86]. Therefore, PTEN inhibition contributes to the progression of fatty liver disease.

Increased ROS levels can harm cytosolic redox-sensitive components via reversible modification, leading to adaptive programming and cell death [87,88]. However, it is necessary to indicate ROS targets participating in ALD pathology. Previous studies have shown that PTEN is oxidatively inactivated in the -SOH state upon H_2_O_2_ exposure, which leads to the formation of an intramolecular disulfide bond between Cys^71^ and Cys^124^ [60,74,75]. Furthermore, there is no evidence showing the mechanisms by which the PTEN redox status is affected by ALD. Our recent study revealed for the first time that the PTEN oxidative status plays a significant role in alcoholic hepatic steatosis [60].

Lipid droplets were found to accumulate around the central vein in the liver when mice were given ethanol for two weeks. Homogenized mice livers showed that PTEN was oxidized upon more prolonged exposure to ethanol [60]. In addition, in vitro experiments with human hepatocellular carcinoma (HepG2) cells and mice primary hepatocytes showed that PTEN was oxidized to the -SOH state when exposed to various doses of ethanol for various periods. This oxidation was reversible starting at 5 min and reverted to the reduced form after 30 min by cellular antioxidant enzymes [60]. A disulfide bond between Cys^71^ and Cys^124^ was also formed when PTEN was inactivated by ethanol. Furthermore, ethanol itself could not induce the oxidation of recombinant PTEN, and pretreatment of cells with *N*-acetyl cysteine, an ROS scavenger, considerably decreased PTEN oxidation levels [60]. These results suggest that ethanol induces PTEN oxidation through its metabolites, especially ROS.

It has been demonstrated that PTEN oxidation was aggravated in the livers of Prx III knockout mice. Furthermore, when cells were treated with rotenone (a mitochondrial complex I inhibitor) and antimycin A (a mitochondrial complex III inhibitor), PTEN oxidation levels were increased compared with those in untreated cells [60]. These findings suggest that ethanol-induced mitochondrial ROS generation contributes, at least partially, to PTEN oxidation.

**Figure 2:**
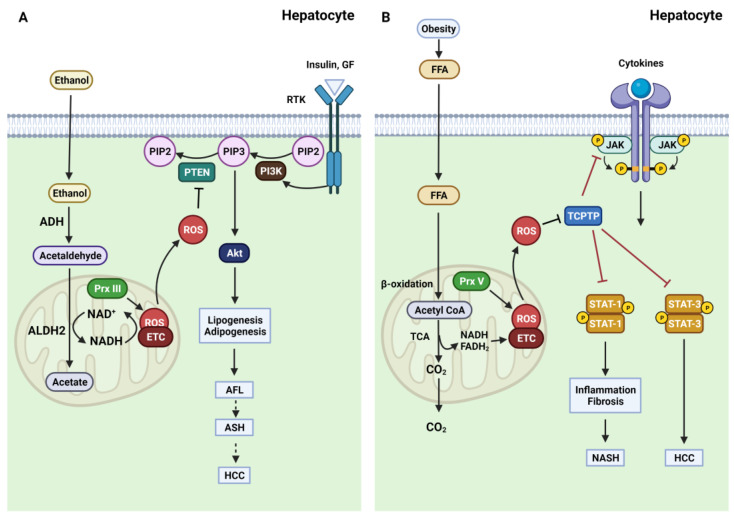
Oxidative inactivation of PTEN in ALD and TCPTP in NAFLD. (**A**). Ethanol is transported into the cytosol of hepatocytes, where ethanol metabolism occurs. Ethanol oxidation into acetaldehyde is primarily responsible for ADH, and this intermediate acetaldehyde product is converted to acetate by ALDH2 in the mitochondria. This conversion generates ROS via ETC-induced mitochondrial dysfunction. ROS overproduction causes oxidative inactivation of PTEN, leading to Akt hyperactivation and, finally, hepatic steatosis. (**B**). FFA arising from HFD-induced obesity is transported to the cytosol. Most FFA is metabolized by β-oxidation in the mitochondria. Increased FFA levels are associated with overloaded β-oxidation and subsequent mitochondrial dysfunction. ROS are overproduced due to elevated electron transfer from NADH and FADH_2_ to the ETC. Enhanced ROS levels induce TCPTP oxidation, activating JAK, STAT-1, and STAT-3 signaling. The hyperactivation of STAT-1 accelerates the development of NASH, while the hyperactivation of STAT-3 triggers HCC.

Research on the PI3K/PTEN/Akt signaling pathway has also shown its involvement in alcohol-induced liver injury [89,90]. Inhibition of PTEN lipid phosphatase activity led to an increase in Akt activity. Akt stimulation was associated with the upregulation of SREBP1c, a key regulator for inducing lipogenic gene expression and promoting FAS. Ethanol exposure increased p-Akt and SREBP1c in vivo and in vitro. Moreover, PPARγ, a key regulator of adipogenesis, also increased, leading to enhanced levels of FAS, a fatty-acid-modifying enzyme that acts downstream of SREBP1c and PPARγ [60]. These findings show the importance of the PTEN redox state in the onset of alcoholic steatosis, indicating that PTEN may be a potential therapeutic target for alcohol-induced liver diseases. In addition, alcoholic steatosis can progress to two advanced stages: ASH and HCC. Therefore, further studies are needed to investigate the redox regulation of PTEN in the progression of ASH and HCC (Figure 2A).

### 3.2. TCPTP and NAFLD

#### 3.2.1. TCPTP and Its Redox Regulation

*PTPN2*, which encodes the TCPTP protein, is located on chromosome 18p11.2–p11.3, which was found in 1992 [91]. Studies have shown that *PTPN2* deletion is characteristic of acute lymphoblastic leukemia and breast cancer, suggesting that TCPTP functions as a tumor suppressor [13,14]. In addition, TCPTP was first cloned from a T-cell cDNA library by referring to it as a classical non-transmembrane protein tyrosine phosphatase [11]. TCPTP exists as two variants, TC45 and TC48, due to alternative splicing events, and their names denote the predicted sizes of 45 and 48 kDa, respectively [92,93]. The structure of both isoforms consists of an N-terminal catalytic domain (residues 1–300) and a C-terminal tail containing a nuclear localization signal (NLS). Therefore, TC45 is located in the nucleus and, in response to various stimuli, such as cytokines or insulin, translocates from the nucleus to the cytoplasm [92,93,94]. However, TC48 contains an additional 3 kDa hydrophobic segment that inhibits the NLS function. TC48 overexpression in the ER is detected by immunofluorescence [92].

TCPTP, a member of the PTP family, regulates various signaling pathways via its tyrosine-specific phosphatase function. TCPTP dephosphorylates various substrates, including receptor tyrosine kinases [95,96], Janus-activated kinase (JAK) 1 and 3 [97,98,99], and signal transducer and activator of transcription (STAT)-1, 3, 5, and 6 [100,101,102,103]. Research has shown that STAT activation regulates lipogenesis, inflammation, and carcinogenesis. STAT-1 is dephosphorylated by TCPTP, leading to the downregulation of interleukin (IL)-6 [104], IL-7 [105], and interferon-γ (IFN-γ) signaling [106]. Similarly, STAT-3 is dephosphorylated at the Y705 site by TCPTP, which downregulates IL-6 and IFN-γ signaling [107,108]. However, hepatic TCPTP deficiency promoted STAT-1, 3, and 5 signaling, which facilitated steatosis in mice fed an HFD for 12 weeks, compared with control mice [109].

It has been shown that PTPs are regulated by ROS, which inhibit their activity. For classical PTPs, such as PTP1B, there is a mechanism to prevent the irreversible oxidation of the nucleophilic cysteine by SO_2_H or SO_3_H [6]. During oxidation, sulfenic acid is rapidly converted to cyclic sulfenamide, formed by the covalent linkage of the sulfur atom of the catalytic Cys^215^ and the nitrogen atom of an adjacent residue. This intermediate causes structural alterations resulting in the inhibition of substrate binding and the exposure of the oxidized cysteine to the cellular environment [6,110]. In addition to protecting against irreversible oxidation, the intermediate sulfenamide is reduced to its active form. TCPTP, a classical PTP, is closely related to PTP1B, with around 72–74% sequence homology in the catalytic domain [95,111]. Research on the structure of TCPTP has revealed superimposed cysteine residues between the Cys^216^ of TCPTP and the Cys^215^ of PTP1B [111]. TCPTP and PTP1B are reversibly oxidized upon insulin-stimulation-induced ROS generation, suggesting that sulfenic acid is converted into cyclic sulfenamide after oxidation (Figure 1B) [112]. Further research on the mechanism of TCPTP oxidation may reveal its nucleophilic cysteine site and how it maintains the regulation of the catalytic function.

The Trx system has been shown to reduce sulfenamide species. Among PTPs that form cyclic sulfenamide during oxidation, PTP1B is effectively reduced by Trx [113]. PTP1B interacted with Trx1 via a mechanism-based trapping approach during the treatment of Hela cells with H_2_O_2_. The interaction was determined by the formation of intermolecular disulfide bonds between Trx and PTP1B when the second cysteine of Trx was mutated to serine (Trx1-C35S) and stabilized this mixed disulfide intermediate [113]. Furthermore, Trx1 overexpression in 293T cells led to the decrease in phosphorylation of the insulin receptor β subunit, in contrast to the increased phosphorylation in cells with Trx1-C35S [113]. These findings suggest that Trx oxidoreductase is important for the reactivation of oxidized PTP1B into its reduced form. However, there are no studies focusing on the reversible reactivation of TCPTP oxidized by the Trx system. However, owing to its oxidoreductase function, which has been confirmed by the reduction in oxidized PTPs, such as PTEN and PTP1B, TCPTP might be a potential candidate for Trx-dependent reversible reactivation upon oxidation by ROS.

#### 3.2.2. Oxidative Inactivation of TCPTP in NAFLD

TCPTP was initially studied as a negative regulator of the insulin signaling [112,114]. TCPTP knockout murine embryo fibroblasts showed an increase in phosphorylation of the insulin receptor β subunit, which further activates the PI3K/Akt signaling pathway [114]. Furthermore, TCPTP was reversibly oxidized by insulin stimulation, and the insulin receptor β subunit was indicated as TCPTP’s substrate [112]. Later research in Gpx1-deficient mice induced an oxidative stress and oxidative inactivation of TCPTP but reduced β cell insulin secretion via the activation of STAT signaling [115]. Therefore, TCPTP inhibition can regulate insulin secretion and improve insulin signaling.

Obesity is a known risk factor for NAFLD, and it increases the levels of ROS and oxidative stress. A previous study demonstrated PTP oxidation in mice fed an HFD (23% fat) [109]. PTPs were extensively oxidized in HFD mice livers, compared with those in chow-fed mice livers, and the PTP oxidation was irreversible due to the -SO_3_H. These oxidized PTPs included PTP1B and TCPTP, which suggests the consequential redox regulation of TCPTP in obesity-induced oxidative stress [109]. The oxidation of TCPTP affected its substrates, such as p-STAT-1 and p-STAT-3, which were enhanced in HFD (24 weeks) mice livers. Notably, hepatic TCPTP deficiency led to the accumulation of lipid droplets, a morphological feature of steatosis, and elevated the levels of liver SREBP1c and PPARγ, enabled by the activation of STAT-1 and STAT-3 signaling [109]. These results demonstrate that the redox regulation of TCPTP is important in promoting hepatic lipogenesis and steatosis by HFD-induced obesity.

As observed in a previous study, PTPs, including TCPTP, were oxidized to -SO_3_H in the HFD mice livers that developed hepatic steatosis. However, the redox regulation of PTPs in the development of NASH and HCC remains unexplored. Different mouse models, including a standard chow diet model, an HFD model (promoting obesity, IR, and simple steatosis, but not NASH), and a choline-deficient HFD model (CD-HFD promoting obesity, IR, and the progression of simple steatosis to NASH), were used to examine whether PTP oxidation was associated with NASH [116]. In particular, the TCPTP oxidation status was more evident in CD-HFD mice livers than in the normal chow diet mice livers. Notably, STAT-1 and STAT-3 phosphorylation was also increased in the CD-HFD mice livers that developed NASH [116]. This finding suggests the potential role of the TCPTP redox status in the progression of NASH, which is mediated by substrates such as STAT-1 and STAT-3.

Mice with TCPTP-deficient hepatocytes (*Alb*-Cre;*Ptpn2^fl/fl^*) were fed an HFD to further investigate the role of TCPTP in NASH and HCC. These mice developed NASH, which involved hepatocyte ballooning; lymphocytic infiltrates, including CD4^+^ and CD8^+^ T cells and immunoglobulin A; and ectopic lymphoid-like structures [116]. Furthermore, a third of the *Alb*-Cre;*Ptpn2^fl/fl^* mice with HFD showed progressive tumors with many characteristics of aggressive HCC. Since both STAT-1 signaling and STAT-3 signaling were enhanced upon TCPTP deletion, the function of these signaling pathways in NASH and HCC was analyzed using mice with TCPTP-deficient hepatocytes fed an HFD. STAT-1 heterozygosity (*Stat-1^fl/+^*) repressed hepatic inflammation, which was indicated by the reduction in STAT-1 target genes such as *Cxcl9* and *Lcn2*. Immune cell recruitment was reduced, as indicated by the decrease in CD4^+^ and CD8^+^ T cells. *Stat-1^fl/+^* also repressed fibrosis in HFD *Alb*-Cre;*Ptpn2^fl/fl^* mice [116]. However, STAT-3 heterozygosity (*Stat-3^fl/+^*) did not reverse the increase in *Cxcl9* or *Lcn2* expression, immune cell recruitment, and fibrosis in mice with TCPTP-deficient hepatocytes. *Stat-3^fl/+^* in HFD-fed *Alb*-Cre;*Ptpn2*^fl/fl^ mice inhibited the development of HCC, unlike the control mice [116]. These findings suggest that NASH and HCC arising from obesity are driven by independent pathways since TCPTP is inactivated by oxidation. TCPTP inactivation contributes to NASH and fibrosis via a STAT-1-dependent pathway, in contrast to HCC, which is promoted by a STAT-3-dependent pathway (Figure 2B) [116]. These results might explain the growing number of patients with HCC without cirrhosis associated with obesity; however, this needs further validation.

## 4. Conclusions and Future Perspectives

ALD and NAFLD are the most common chronic liver diseases, contributing to the high mortality of liver cirrhosis and HCC. Numerous studies have focused on fatty liver pathogenesis; however, this process is not fully understood. Since patients with early disease stages, such as steatosis and low and moderate steatohepatitis, are often asymptomatic, early diagnosis is difficult and sometimes accidental. Therefore, it is crucial to fully understand the pathogenesis of this condition in order to to discover specific potential molecules for diagnosis and treatment.

In ALD and NAFLD pathology, oxidative stress via ROS overproduction plays an indispensable role, resulting in oxidative damage to DNA, lipids, and proteins. Mitochondrial dysfunction is considered the leading cause of ROS overproduction in these diseases. Once mitochondrial function decreases, abnormal generation of ROS increases, producing a vicious cycle between oxidation-induced liver damage and mitochondrial deterioration. Prx deficiency facilitates mitochondrial-ROS-induced liver injury. One of the harmful effects of oxidative-stress-induced ROS overproduction is the modulation of redox-sensitive components via reversible oxidation. PTPs such as PTEN and TCPTP are primary targets of ROS, which inhibit PTP activity via oxidation. PTEN favors the disulfide bond between Cys^71^ and Cys^124^, whereas TCPTP forms cyclic sulfenamide upon oxidation by ROS. Redox regulation of PTEN and TCPTP is involved in various cell signaling and biological processes including insulin signaling [112,117], growth factor signaling [109,118], muscle differentiation [119], and immunity [120].

Our recent study on alcoholic steatosis demonstrated that PTEN was reversibly oxidized (-SOH) when exposed to ethanol in vivo and in vitro. In addition, the deficiency of Prx III, a mitochondrial ROS scavenger, increased the oxidation of PTEN and lipid accumulation, which emphasized the importance of increased mitochondrial ROS generation in the progression of PTEN-mediated hepatic alcoholic steatosis. Further research, in which redox regulation of PTEN is well examined, is needed to investigate more advanced stages of ALD, such as ASH or HCC. Additionally, studies have shown that TCPTP was inactivated and oxidized to the irreversible -SO_3_H state in the livers of obesity-induced NAFLD mice. This inactivation was accompanied by the triggering of TCPTP substrates, including STAT-1 and STAT-3. STAT-1 and STAT-3 signaling promoted separate pathways for the development of NASH and HCC, respectively. These studies determined the mechanism by which HCC cases developed without advanced cirrhosis or fibrosis. Furthermore, since oxidative-stress-induced ROS overproduction is common in ALD and NAFLD, the PTEN redox status in NAFLD and the TCPTP redox status in ALD should be cross-checked in the future.

Several studies showed that inhibiting Akt, downstream of PTEN, or STAT, downstream of TCPTP, could be potential treatments for fatty liver disease. For instance, mice were treated with an Akt inhibitor, MK-2206, that prevented NAFLD and liver cancer progression [121]. MK-2206 has been used in several clinical trials either as a monotherapy or in a combination with chemotherapy to treat solid tumors and cancers [122,123,124,125,126,127]. MK-2206 showed an acceptable toxicology profile and could be a clinical safety drug [122,123,124]. Although MK-2206 monotherapy had limited clinical activity in advanced breast cancer and uterine serous carcinoma [125,126,127], it is necessary to investigate the effects in fatty liver disease. The interaction between miR-149 and Akt1 prevented HCC tumorigenesis in vitro, and patients with low miR-149 expression had decreased overall survival and disease-free survival rates [128]. C188-9, a STAT-3 inhibitor, improved NASH and blocked HCC growth in hepatocyte-specific deletion of PTEN, highlighting C188-9 as a potential drug for the treatment and prevention of HCC [129]. C188-9 is also using as a monotherapy for patients with advanced solid tumors, including HCC in a phase I clinical trial (NCT03195699), and the outcome will be updated in the future. In addition, the role of antioxidants is also mentioned in the treatment of fatty liver disease. A study has shown that Trx1 treatment by daily intraperitoneal injection in atherogenic high-fat diet mice protected the liver from atherosclerosis-associated liver steatosis represented by decreased liver weight, liver TG, and mRNA levels of SREBP1-c and PPARα [130]. In summary, the pathogenic contributions of PTEN and TCPTP might open new directions for treating fatty liver disease either by targeting their downstream signaling pathways or controlling redox homeostasis.

## Abbreviations

4-HNE	4-Hydroxylnonenal
ADH	Alcohol dehydrogenase
ALD	Alcoholic liver disease
ALDH2	Aldehyde dehydrogenase
AMPK	Adenosine monophosphate-activated protein kinase
aP2	Adipocyte protein 2
CD-HFD	Choline-deficient high-fat diet
CEBPA	CCAAT-enhancer-binding protein alpha
CP	Peroxidatic cysteine
CR	Resolving cysteine
CREB1	cAMP-responsive element-binding protein 1
c-Src	Proto-oncogene tyrosine-protein kinase Src
CYP2E1	Cytochrome P450 2E1
ER	Endoplasmic reticulum
ETC	Electron transport chain
FAK1	Focal adhesion kinase 1
FAO	Fatty acid oxidation
FAS	Fatty acid synthesis
FFA	Free fatty acid
GLUT4	Glucose transporter type 4
Gpx	Glutathione peroxidase
Grx	Glutaredoxin
GSH	Glutathione
HCC	Hepatocellular carcinoma
HFD	High-fat diet
IFN-γ	Interferon gamma
IL-6	Interleukin-6
IL-7	Interleukin-7
IR	Insulin resistance
IRS-1	Insulin receptor substrate-1
JAK	Janus activated kinases
MEOS	Microsomal ethanol oxidation system
miR-21	microRNA-21
MTS	Mitochondrial targeting signal
NAFLD	Nonalcoholic fatty liver disease
NASH	Nonacoholic steatohepatitis
NLS	Nuclear localization signal
NOX	Nicotinamide adenine dinucleotide phosphatase oxidase
O_2_^•−^	Superoxide anion radicals
HO^•^	Hydroxyl radicals
PIP2	Phosphatidylinositol (4,5)-bisphosphate
PIP3	Phosphatidylinositol (3,4,5)-trisphosphate
PPARγ	Peroxisome proliferator-activated receptor gamma
Prx	Peroxiredoxin
PTEN	Phosphatase and tensin homolog deleted on chromosome 10
PTP1B	Protein tyrosine phosphatase 1B
PTPs	Protein tyrosine phosphatases
PTPN2	Protein tyrosine phosphatase nonreceptor 2
ROS	Reactive oxygen species
-SOH	Sulphenic acid
-SO_2_H	Sulfinic acid
-SO_3_H	Sulfonic acid
SREBP1	Sterol regulatory element-binding protein 1
STAT	Transducer and activator of transcription
STAT-1^fl/+^	STAT-1 heterozygosity
STAT-3^fl/+^	STAT-3 heterozygosity
TCPTP	T cell protein tyrosine phosphatase
Trx	Thioredoxin
TrxR	Thioredoxin reductase
